# Valorization of Artichoke Industrial By-Products Using Green Extraction Technologies: Formulation of Hydrogels in Combination with Paulownia Extracts

**DOI:** 10.3390/molecules26144386

**Published:** 2021-07-20

**Authors:** Gabriela Órbenes, Paula Rodríguez-Seoane, María Dolores Torres, Rolando Chamy, María Elvira Zúñiga, Herminia Domínguez

**Affiliations:** 1 Escuela de Ingeniería Bioquímica, Pontificia Universidad Católica de Valparaíso, Avenida Brasil 2085, Valparaíso 2340950, Chile; gabriela.orbenes.v@mail.pucv.cl (G.Ó.); rchamy@nbcpucv.cl (R.C.); 2 Centro Regional de Estudios en Alimentos Saludables, CREAS, Av. Universidad 330, Curauma, Valparaíso 2340950, Chile; maria.zuniga@pucv.cl; 3 Departamento de Enxeñería Quimica, Universidade de Vigo (Campus Ourense), Edificio Politécnico, As Lagoas, 32004 Ourense, Spain; matorres@uvigo.es (M.D.T.); herminia@uvigo.es (H.D.)

**Keywords:** microwave, autohydrolysis, rheometry, antiradical properties, saccharidic fraction

## Abstract

The integral valorization of artichoke bracts generated during industrial canning of artichoke was assessed. The extraction of bioactive compounds was addressed with pressurized hot water under subcritical conditions. The performance of this stage on the extraction of phenolics with antioxidant properties and the saccharidic fraction using conventional and microwave heating was compared. The microwave assisted process was more efficient than the conventional one regarding extraction yields of total solubles, and glucose and fructose oligomers and phenolics, because lower operational temperatures and shorter times were needed. Degradation of fructose oligomers was observed at temperatures higher than 160 °C, whereas the maximal phenolic content occurred at 220 °C. Both the extracts and the residual solids, obtained at conditions leading to maximum phenolics yields, were evaluated for the production of starch-based hydrogels, supplemented with Paulownia leaves’ aqueous extracts.

## 1. Introduction

The agri-food industry generates millions of tons of waste each year, leading to serious environmental problems and a loss of valuable compounds [1]. Artichoke (*Cynara scolymus* L.) is used as food and as a traditional remedy [2,3], and large amounts of waste (about 80–85% of the total plant biomass) are generated during industrial processing. Over the last years, attempts have been made to find possible uses for artichoke by-products (bracts, leaves, and stems) [4]. These by-products are a source of fatty acids [5], inulin, fiber, minerals [6], inositols [7], and phenolic compounds [2,6,7,8,9,10,11], suitable for the production of dietary supplements and food additives.

Methanol, ethanol, and water have been used for the extraction of sugar and phenolics from bracts [3,5,8,9]. Ultrasound-assisted extraction with water [12] and also with 60% methanol [6] enhanced the extraction of phenolics and prebiotic fructans [13]. Microwave assisted extraction and pressurized extraction with water have been used for the extraction of inositols and inulin [14] or caffeoylquinic acids [7]. Pressurized hot water extraction was used to extract caffeoylquinic acids and flavone glycosides without formation of artefacts at high temperatures [10].

Both techniques, subcritical water extraction (or autohydrolysis) and microwave assisted extraction, are efficient and promising methods for recovering natural compounds from raw plants or their by-products. Subcritical water refers to water at temperatures ranging from the boiling point (100 °C) to critical point (374 °C), and pressure high enough to maintain the liquid state. Under these conditions, water has unique properties, such as lower dielectric constant, better diffusion, and higher ionization constant [15,16]. Microwave extraction combines microwave energy and traditional solvents, with the difference being that the energy is used to heat solvents in contact with samples, and the direction of heating occurs from the inside to the outside. In microwave extraction, the extraction time is significantly reduced in comparison with classical extraction techniques [17].

Polyphenols are gaining more attention due to their therapeutic effects and their potential technological applications (food, pharmacy, materials engineering, and fine chemistry). Due to their antioxidant profile, phenolic compounds can be used for food preservation and for preparing bioactive packaging, elaboration of hydrogels and nanocomplexes, in addition to improving properties of starch [18]. Starch, which is widely used as carrier for antioxidant or antimicrobial agents [19,20], can be obtained from various plants, such as cassava, rice, potato, wheat, and maize. Starch-based biomaterials are used in food packaging and edible film applications due to their abundance, biodegradability, low cost of production, and ease of use in film preparation [19,20]. Starch does not have inherent antimicrobial and antioxidant properties, so it is necessary to provide other materials with these properties; agro-industrial and forest wastes being a cheap source. The leaves from the rapid growth tree Paulownia are a residue from plantations and contain bioactives with antibacterial, antioxidant, and anti-inflammatory activities [21].

The main objective of this work is the valorization of industrial artichoke by-products. For this purpose, extraction with pressurized hot water under subcritical conditions operating with conventional heating (CH) and with microwave assisted heating (MAH) were compared. The extracts produced under optimal conditions and the remaining residual solids were used for the preparation of functional starch-based hydrogels, which were also supplemented with Paulownia leaves’ extracts obtained by autohydrolysis.

## 2. Results and Discussion

### 2.1. Artichoke Wastes Characterization

**Figure 1:**
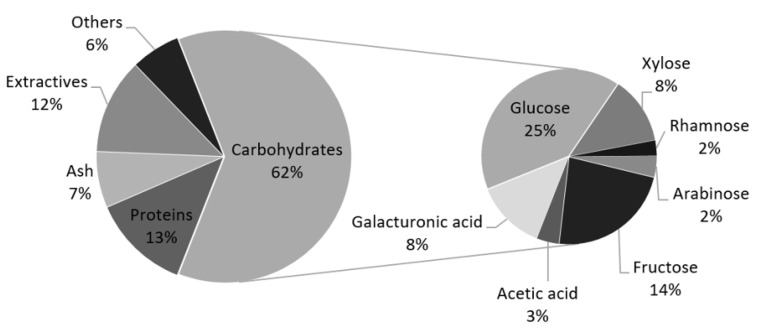
Proximal composition of artichoke bracts.

Proximal composition of artichoke bracts is shown in Figure 1. Protein content was 12.55 ± 0.08%, in agreement with previous findings reporting values in the range 10.25–15.2% [11,22]. The ash content determined was 7.12 ± 0.04%, in accordance with content reported by Lutz et al. [23] in the range 6.7–7.6%. The content in ethanol extractives accounted for up to 12%, and carbohydrates accounted for 62%. A higher carbohydrate content (75%) was presented in other studies [23,24], but it was calculated by difference of the sum of other components excluding extractives.

The information of carbohydrates found for artichoke bracts is limited, but Pesce et al. [25] identified in the “Spinoso sardo” artichoke genotype, 23.62% glucans and 17.70% hemicelluloses composed by 12.57% xylose, 3.35% arabinose and 1.79% acetic acid, comparable to the values reported in this work.

### 2.2. Extraction of Bioactive Fractions

Two different extraction methods of autohydrolysis heating were tested to compare the phenolic extraction, antiradical properties, and saccharidic fraction of the extracts.

**Figure 2:**
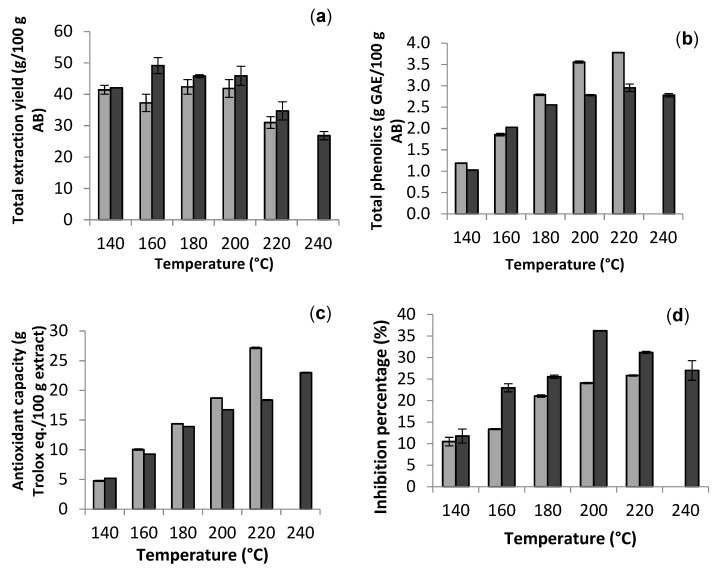
Comparison between autohydrolysis with conventional heating (dark gray) and microwave heating (light gray) on the extraction yield of (**a**) total solubles, (**b**) total phenolics, (**c**) ABTS radical scavenging capacity, and (**d**) DPPH inhibition percentage of the extracts.

Maximal extraction yield from artichoke bracts during conventional heating was achieved operating at 160 °C, whereas no significant differences were observed in the range of 140–200 °C with microwave assisted process (Figure 2a). The microwave assisted operation allowed the extraction of higher amounts of total phenolics (Figure 2b) than the conventional one. Regardless of the heating strategy, an increase in severity led to increased phenolic yields at 220 °C, showing a maximum of 2.9 and 3.8 g GAE/100 g artichoke bracts (AB) in conventional and microwave heated equipment (Figure 2b), respectively. The phenolic content ranged from 2 to 12 g/100 g extract, and compared favorably with those reported for alcoholic extracts [6,7]. Similar behavior was observed for the antiradical properties determined by ABTS scavenging (Figure 2c) and expressed in g Trolox eq./100 g extract, with maximums of 22.9% in conventional extraction and 27.2% in microwave heating at the highest temperature, comparable or superior to the potency reported for ethanolic extracts [6,8]. DPPH radical scavenging capacity of the extracts diluted 1:50 were expressed as inhibition percentage and showed better results in conventional heating extraction, reaching 36.2% versus 25.8%. TEAC values higher than 20 g Trolox eq./100 g extract were observed at temperatures higher than 200 °C, and the maximum in DPPH radical scavenging was observed at that temperature in conventional extraction.

In a previous study, the extraction temperature was found in the content of caffeoyl quinic compounds. Whereas 1,5-diCQA was degraded with increased temperature, cynarin (1,3-diCQA) was formed at temperatures above 110 °C, due to isomerization reactions [10]. Usually, at high temperatures, the plant tissues are softened, and therefore the cell wall membranes too. This effect results in a facilitated diffusion of phenolic compounds into the solvent. However, it should be taken into account that prolonged exposure time could decrease the extraction yield due to the oxidation and degradation of the target compounds. In addition, the formation of undesirable compounds may be promoted. A balance between applied extraction time and temperature is needed [16,26].

Both heating strategies provided extracts with fructose as the major monomer, with a maximum yield of 12 g/100 g AB at 180 °C, representing 86% of the total content, followed by glucose with a maximum of 5.5 g/100 g AB at the same temperature.

**Figure 3:**
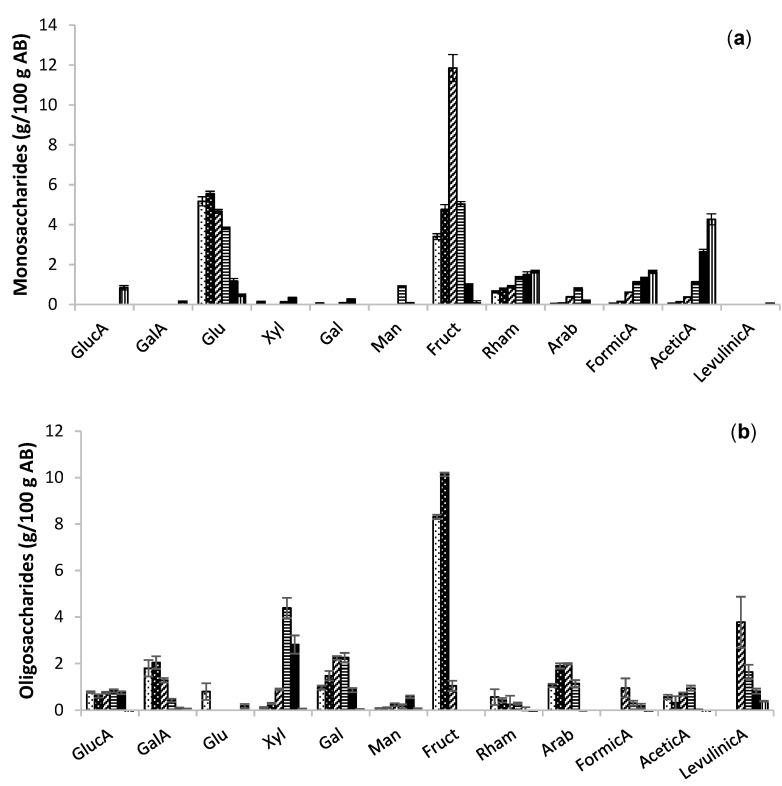
Extraction of (**a**) monosaccharidic and (**b**) oligosaccharidic fractions of extracts obtained at different temperatures; 140 (black dots), 160 (white dots), 180 (slanted lines), 200 (horizontal lines), 220 (filled bars), and 240 °C (vertical lines) of conventional heating.

**Figure 4:**
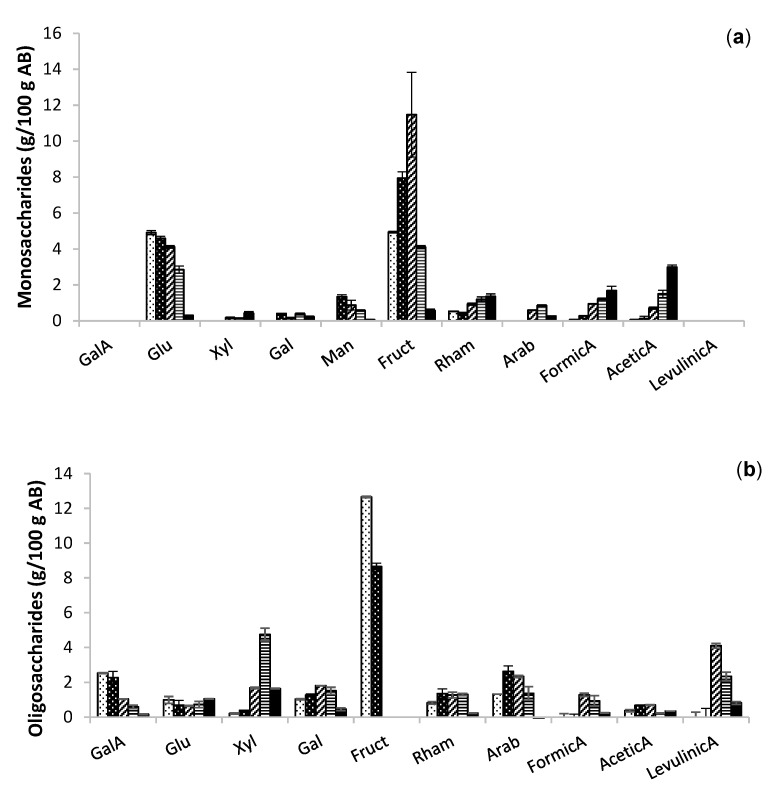
Extraction of (**a**) monosaccharidic and (**b**) oligosaccharidic fractions of extracts obtained at different temperatures; 140 (black dots), 160 (white dots), 180 (slanted lines), 200 (horizontal lines), and 220 °C (filled bars) of microwave assisted heating.

Monomers were more abundant in the extracts obtained at 180 °C and the organic acids, increasing in severity at 200–240 °C. The monomeric concentration was similar in both technologies (Figure 3a and Figure 4a), but the oligomer contents in the autohydrolysis (Figure 3b) extracts were slightly higher with the microwave assisted technology (Figure 4b). The yield of oligomers of fructose was slightly higher in microwave than in conventional heating, with maximum values of 12.66 g/100 g AB at 140 °C and 10.14 g/100 g AB at 160 °C. Oligomers of fructose were not detected at temperatures higher than 180 °C. The dramatic decrease in fructose with increasing temperatures was also expected [14].

### 2.3. Rheology of the Formulated Hydrogels

**Figure 5:**
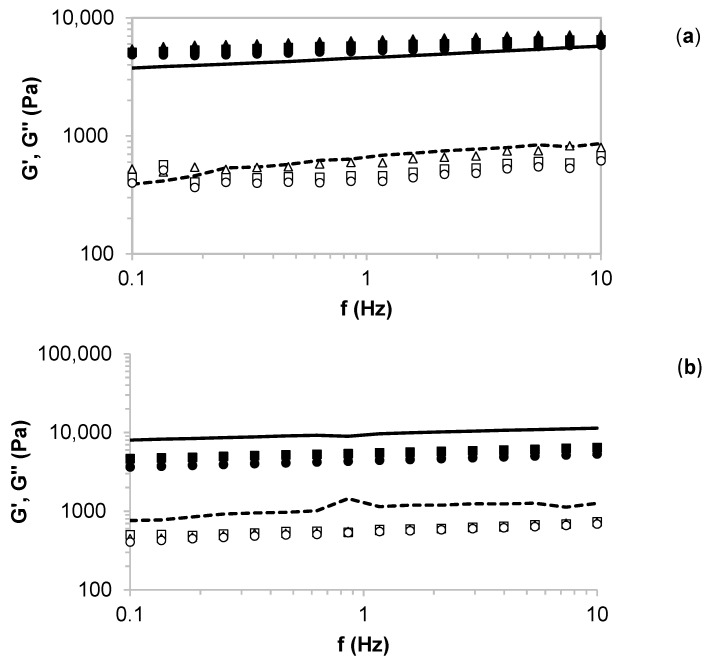
Impact of the extraction treatment of artichoke bracts ((**a**), conventional and (**b**), microwave) on the oscillatory measurements of starch-based hydrogels formulated with autohydrolysis extracts of artichoke and Paulownia leaves at different volume ratios. Symbols: G’ (closed), G” (open), 25:75 (squares), 50:50 (triangles), 75:25 (circles), and 100:0 (lines).

Figure 5 presents the effect of (a) conventional and (b) microwave-assisted heating during production of artichoke bracts extracts on the viscoelastic behavior of the starch-based matrices prepared with the corresponding artichoke and Paulownia extracts at different ratios. For both treatments, elastic (G’) and viscous (G”) moduli were almost frequency independent over the tested frequency range. In all cases, elastic modulus was larger (about 10-fold) than the viscous one at a fixed frequency, which is indicative of typical gel behavior, as previously reported elsewhere for similar matrices [27]. Concerning magnitudes of both moduli, hydrogels formulated with microwave extracts in the absence of Paulownia (100:0) exhibited higher G’ and G” values (around 2-fold) than those prepared with their autohydrolysis counterparts. This behavior is consistent with the aforementioned extraction yields (Figure 3a), suggesting higher water competition between the gelling biopolymer and antioxidant extracts. The incorporation of microwave treated Paulownia extracts at different tested ratios involved a decrease in the viscoelastic behavior of the starch-based hydrogels when compared with artichoke samples. The highest softening of the gels was identified for systems prepared at the ratio of 75:25. In contrast, the addition of soluble extracts of Paulownia treated by autohydrolysis promoted the strengthening of the elastic behavior of starch-based hydrogels, while it entailed a weaking of the viscous behavior. In this case, the highest strength was observed for samples made at a 25:75 ratio. This agrees with the antioxidant trends explained above (Figure 3b–d) and highlights the relevance in terms of processing and the final application of the understanding of the impact of different types of extraction, and formulation composition, on the rheological behavior of the corresponding gelling matrices. All formulated hydrogels featured intermediate gel characteristics, consistent with those previously found for other similar biopolymer-based gels, incorporated with antioxidant soluble extracts from natural underused materials [27,28].

**Figure 6:**
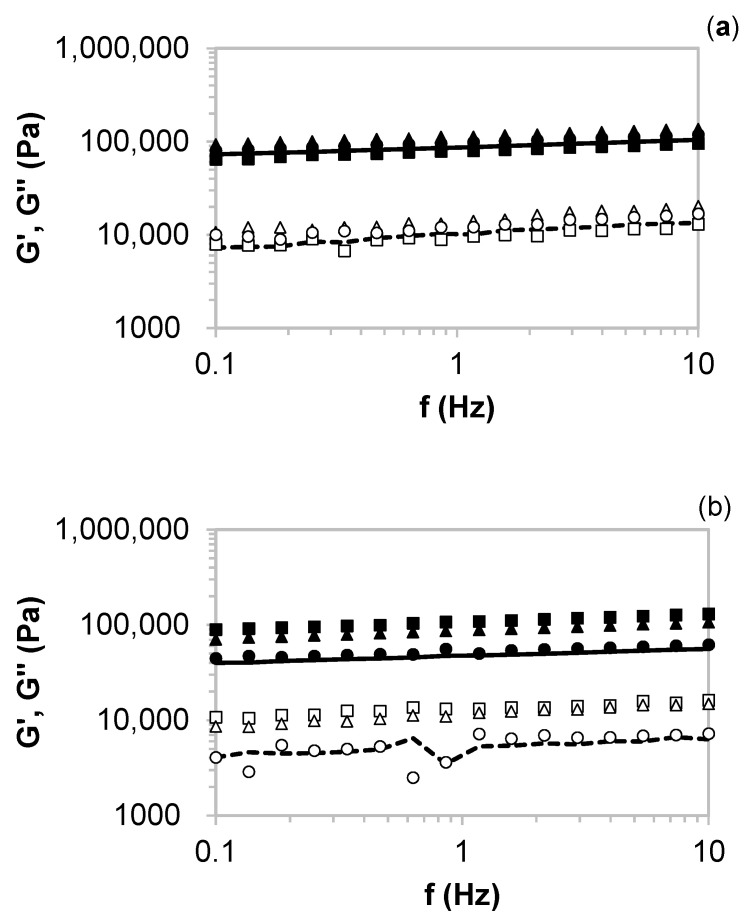
Oscillatory measurements of starch-based hydrogels formulated with the solid residues from artichoke bracts obtained with different techniques ((**a**), conventional and (**b**), microwave) and mixed with Paulownia leaves’ autohydrolysis extracts at different ratios. Symbols: G’ (closed), G” (open), 25:75 (squares), 50:50 (triangles), 75:25 (circles), and 100:0 (lines).

Figure 6 shows the viscoelastic profiles for starch-based hydrogels formulated with the solid residue remnants after (a) conventional and (b) microwave heating of artichoke and Paulownia samples. All samples exhibited characteristic gel behavior, although with marked differences between those prepared with extracts from different extraction technologies. No noticeable differences in both moduli were identified between the hydrogels made with the solid residues from autohydrolysis treatment. Those made with microwave solid disposals enhanced the starch-based hydrogels in the presence of Paulownia, obtaining the stronger gels for samples prepared at a 25:75 ratio. An increase in the antioxidant Paulownia fraction implied a softening of the starchy matrices, which agrees with the results previously reported for other functional gels [28,29]. Latter author explained the variation in the viscoelastic features of the gels, taking into account the competition from free water between the biopolymer-based matrix and the soluble antioxidant extracts. Stronger hydrogels (about 10-fold) were obtained when compared with those prepared with the soluble extracts. Higher values of the viscoelastic moduli were reported for other starchy matrices where higher particle sizes were involved [30]. Regarding mechanical features, hydrogels made with soluble extracts could be attractive alternatives as a formulations basis for populations with specific requirements [31], such as puree based baby or elders’ food. Additionally, it could be interesting for viscoelastic values to be used as films in the packing industry [32]. Starch-based hydrogels formulated with solid disposals could be healthy options for the development of innovative cosmetics or personal care products, as bases for body scrubs or face masks [33]. A relevant advantage of the prepared hydrogels was the absence of water syneresis after half a month of cold storage.

## 3. Materials and Methods

### 3.1. Material

Bracts of artichoke used in this work were provided by the Pentzke cannery, from the plant located in San Felipe (Valparaíso, Chile). For industrial elaboration, only the receptacle, commonly known as the heart, was required. The bracts (leaves) were removed and discarded as waste. Artichoke bracts were supplied dehydrated and powdered.

### 3.2. Extraction Method

Artichoke by-products were extracted by autohydrolysis using two different heating methods: conventional heating (CH) and microwave-assisted heating (MAH). Autohydrolysis was performed in a 600 mL extractor (Parr Instrument Company, Moline, IL, USA) and in a microwave-assisted extractor (Anton Parr GmbH Monowave 450 Series, Graz, Austria). Milled artichoke bracts were contacted with distilled water in a liquid to solid mass ratio (LSR) (*w*:*w*) of 8:1 for CH and 10:1 for MAH.

In the conventionally heated equipment, autohydrolysis was performed under non-isothermal conditions, up to a heating temperature in the range of 140–240 °C. Then, the suspension was immediately cooled with water through a stainless-steel coil. In microwave assisted autohydrolysis, a heating time of 3 min was established to reach the desired treatment temperature (140–220 °C). Once the selected temperature was reached, isothermal conditions were maintained for 5 min, and finally, the system was cooled. Operation at 240 °C was not feasible with this equipment. In both systems, after extraction, filtration with a vacuum pump was carried out to separate solid and liquid phases.

### 3.3. Analytical Techniques

Moisture content was determined gravimetrically by oven-drying (105 °C) until a constant weight (24–48 h) was reached. Ash content was also determined gravimetrically after calcination in a muffle furnace for 6 h at 575 °C. Likewise, the ethanol extractives were estimated after reflux for 6 h in a Soxhlet. Total nitrogen assay was conducted on a FlashEA 1112 Elemental analyzer (Thermo, Waltham, MA, USA) with Helium (130 mL/min) as the carrier and reference gas (100 mL/min). The temperatures of the oxidation and reduction ovens were 900 °C and 680 °C, respectively, and the oxygen flow was 250 mL/min. Protein content was calculated using the universal conversion factor 6.25 [34].

Carbohydrates of artichoke bracts were determined after acid hydrolysis with 72% sulfuric acid (30 °C, 1 h) and with 4% sulfuric acid (121 °C, 60 min), and then analyzed in a HPLC (Model 1200, Agilent Technologies, Santa Clara, CA, USA) using the following columns: Aminex HPX-87H (50 °C, 0.6 mL/min 0.003 M H_2_SO_4_) and HPX-87P (80 °C, 0.4 mL/min ultra-pure water). The fructose monosaccharide content was determined by diluted acid hydrolysis (DAH) using the following conditions: LSR 15 (*w*:*w*), 0.5% H_2_SO_4_, 60 min, and 121 °C, according to the method of Pesce et al. [25]. Monosaccharides were determined by HPLC analysis of the liquor obtained directly after extraction. For the quantification of oligosaccharides, a posthydrolysis (4% H_2_SO_4_, 121 °C and 40 min) of extracts was carried out. Then, liquors obtained were analyzed by HPLC. External standards were used.

Total phenolic content was determined by the Folin–Ciocalteu method with gallic acid as the standard [35]. Briefly, 0.5 mL of extract was mixed with 3.75 mL of water, 0.25 mL of Folin–Ciocalteu’s reagent (diluted 1:1 with water), and 0.5 mL of sodium carbonate (10%, *w*/*v*). Samples were incubated for 1 h in absence of light at room temperature before absorbance readings at 765 nm. The radical scavenging was determined against 2,2-diphenyl-1-picrilhydrazil (DPPH) [36]. The inhibition percentage was calculated as the relative decrease in absorbance of the extract (50 µL) at 515 nm after 16 min in a 3.6 × 10^−5^ M methanolic solution of DPPH (2 mL). The 2,2′-azinobis-(3-ethyl-benzothiazoline-6-sulfonate) (ABTS) radical scavenging capacity [37] was expressed as Trolox equivalents. Absorbance readings at 734 nm were recorded after 6 min at 30 °C for a mixture of diluted radical solution (1.0 mL) and extract (10 μL). The diluted radical solution was composed by a mixture between ABTS^+^ reagent (0.3840 g of ABTS and 0.0662 g of potassium persulfate) and phosphate buffered saline (PBS). The mixture was ready to use when an absorbance of 0.700 was reached. All assays were determined in triplicate.

### 3.4. Hydrogels: Formulation and Rheology

A first batch of hydrogels was formulated with soluble extracts of artichoke and Paulownia at different ratios (25:75, 50:50, 75:25, 100:0) (*v*:*v*). Starch (15% *w*/*w*) extracted from Agria variety potato was used as the base of the gelling matrix [38]. A second batch of hydrogels were composed of the remaining solid residues (150 µm) after conventional or microwave-assisted autohydrolysis (7%), Agria potato starch (15%) and the ratios above indicated for both soluble extracts. The processing temperatures selected for artichoke solid residues were established, taking into account the antioxidant features determined in this work. Namely, the hydrogels were elaborated with extracts obtained by conventional heating extraction up to 200 °C and by microwave heating extraction at 220 °C. Paulownia leaves were extracted by autohydrolysis under non-isothermal conditions at 240 °C to produce an extract with 19.54 g GAE/100 g extract [39].

In all cases, the different components were mixed and heated for 10 min at 90 °C with constant stirring, following the procedure previously reported [40]. Then, samples were cooled down at room temperature for 1 h and placed in a fridge overnight to promote full hydrogels maturation before rheological testing. All hydrogels were equilibrated at room temperature for 1 h before mechanical measurements.

Rheology of the above hydrogels was evaluated at least in triplicate using small amplitude oscillatory shear measurements. Viscoelastic monitoring of the storage modulus or elastic modulus (G’) and the loss or viscous modulus (G”) was performed on a controlled-stress rheometer (MCR302, Anton Paar Physica, Graz, Austria). The selected measuring geometry was a sand blasted plate-plate (25 mm diameter), which prevented the possible slippage of the hydrogels. Before rheological tests, hydrogels were placed between the plates (0.5 mm gap) and rested for 5 min to favor the thermal and structural equilibration. Subsequently, stress sweep tests were conducted from 0.1 to 100 Pa at 1 Hz and 25 °C to determine the linear viscoelastic region (<30 Pa). Then, oscillatory measurements were run at the same temperature from 0.1 to 10 Hz at 15 Pa.

### 3.5. Statistical Analysis

The data sets were analyzed using one-factor analysis of variance. Scheffé test was made to differentiate mean values with a 95% confidence (*p* < 0.05) when differences among means were observed. PASW Statistics v.22 software (IBM SPSS Statistics, Armonk, NY, USA) was used to the statistical analysis.

## 4. Conclusions

Artichoke bracts are a good source of bioactives. For this reason, it is necessary to propose extraction methods to valorize these wastes and to take advantage of their interesting compounds. The optimal temperature extraction was 220 °C, yielding 3.8 g GAE/100 g AB of total phenol content. The antioxidant capacity against ABTS radical was 8.4 g Trolox eq./100 g AB and a 25.8% inhibition percentage. A wide range of gel strengths was found depending on the extraction technique, and the extract ratios used during the gels’ development. Suitable mechanical properties were also identified for hydrogels made with the solid residues from both eco-friendly extraction treatments, with a notable effect due to the presence of Paulownia leaves autohydrolysis extracts. Another advantage from the scientific and industrial point of view is the absence of water syneresis in the formulated matrices. Overall, the proposed formulations play a key role to an integral valorization of underused materials.

## Data Availability

The datasets generated during and/or analysed during the current study are available from the corresponding author on reasonable request.
